# Evaluation of antimicrobial orders with expanded indication for use

**DOI:** 10.1017/ash.2026.10403

**Published:** 2026-06-01

**Authors:** Michael Carlone, Jill Wesolowski, Samantha Loutzenheiser, Chanda Mullen, Steven Gordon, Thomas Fraser, Andrea M. Pallotta

**Affiliations:** Cleveland Clinic Main Campus Hospital: Cleveland Clinic, Cleveland, USA

## Abstract

**Objective:**

To evaluate the impact of requiring infectious source selection on systemic antimicrobial orders for stewardship tracking.

**Design and Setting:**

Multicenter, health system quality improvement project across 17 hospitals and 21 emergency departments.

**Methods:**

All inpatient and ED antimicrobial orders with an indication between September 1 and September 30, 2024 were analyzed. Primary outcome: Prevalence of selected infectious sources. Secondary outcomes: indication frequency, free-text use, and concordance between selected and documented sources in 500 random orders.

**Results:**

Among 57,118 orders, empiric indication was most common (71%). Top sources: respiratory (23%), urinary/renal (19%), skin/soft tissue (16%). Concordance was 95%. Free-text entries (2.6%) often matched existing options; dental/oral emerged as a new category.

**Conclusion:**

Infectious source selection was evaluated and supports future targeted stewardship interventions.

## Background

In 2019, the Centers for Disease Control and Prevention (CDC) developed the Core Elements of Hospital Antibiotic Stewardship Programs with 7 topics: Leadership Commitment, Accountability, Drug Expertise, Action, Tracking, Reporting, and Education.^1,2^ Including an indication or infection source on medication orders can improve communication between caregivers and patients, prevent medication errors, and add structure to antimicrobial stewardship (ASP) activities such as prospective audit and feedback, duration-of-therapy assessment, and timely therapy modification.^3,4^ As part of an ASP program, indication or infectious source can help promote guideline-concordant treatment and minimizes unnecessary antimicrobial exposure.^1^ Omission of indication or infectious source leaves pharmacists to evaluate orders for all possible diseases by chart review with increased effort of the ASP team to identify intervention opportunities and track antimicrobial use.^2^

**Figure 1.**
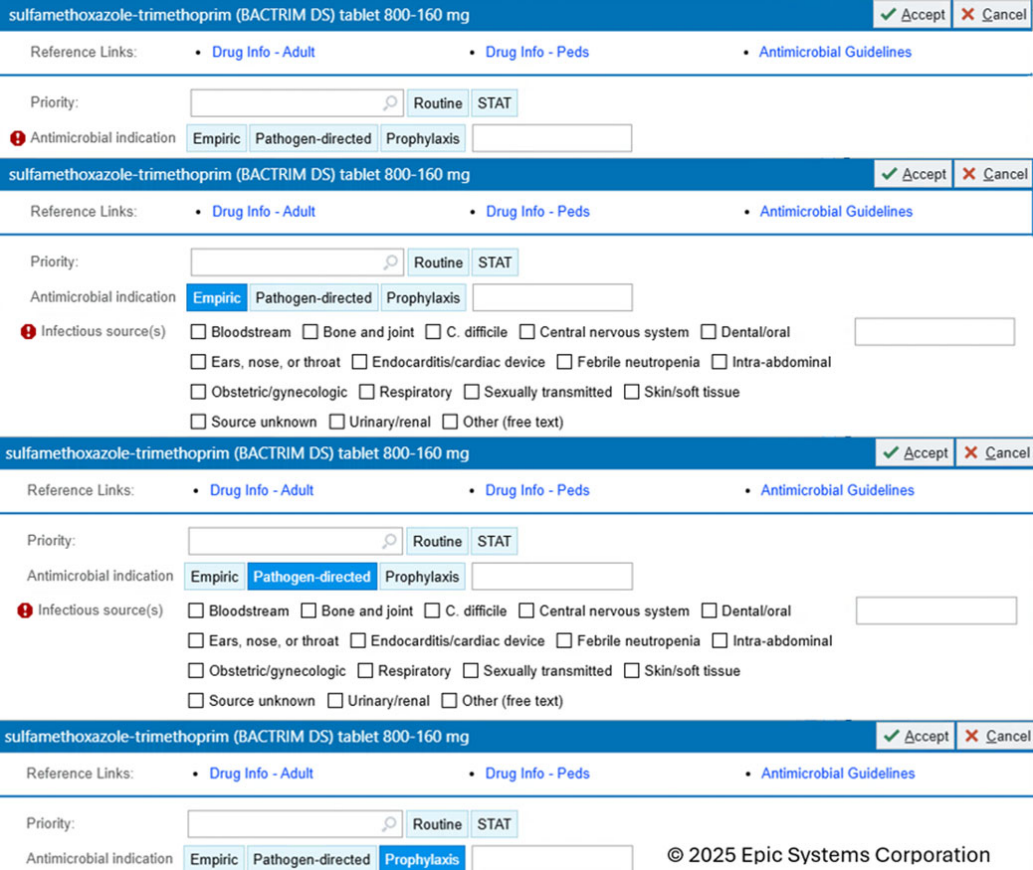
Electronic health record includes a hard stop requiring provider to choose infectious source if “Empiric” or “Pathogen-directed” are chosen.

The ASP Committee implemented mandatory cascading order questions for indication and infectious source on inpatient, systemic antimicrobial orders with intentions to create purposeful communication among caregivers and foster intentionality in provider antibiotic choice (Figure 1). This quality improvement project reported the descriptive pharmacoepidemiology of antimicrobial infectious sources at our health system. The objective described the number of infectious sources utilized in antimicrobial orders and evaluated concordance between infectious source selection based on chart documentation.

## Methods

This was a health system, multicenter quality improvement assessment of antimicrobial orders at 17 hospitals and 21 emergency departments. Following approvals through medical staff pharmacy and therapeutics committee, pharmacy clinical integration committee, nursing practice council, and technical leadership, the indication order question was implemented on 9/20/2016 and infectious source order question was implemented on 6/20/2024. Figure 1 illustrates the Epic® electronic health record (EHR) ordering interface, where providers first select an antimicrobial indication order question (prophylaxis, empiric, or pathogen-directed). Selection of empiric or pathogen-directed indication triggers a hard stop, requiring the provider to answer the cascading infectious source order question (ie, respiratory, urinary tract, etc.) before the order can be completed. If “other” was selected, the prescriber was required to free-text the source. Indication and infectious source order questions were preselected on order sets. Prophylaxis indication selection did not require infectious source selection. Education to prescribers and nurses occurred in staff meetings, newsletters, and intranet stories at all hospitals.

The inclusion criteria were inpatient and emergency department systemic antimicrobial orders with an infectious source selected and at least one dose administered between September 1 and September 30 2024. Order sets were excluded from the analysis because infectious source is preselected. The primary outcome was prevalence of infectious source chosen by the provider.

Secondary outcomes evaluated the utilization of “other (free-text)” infectious source to determine if new infectious source buttons should be added. Additionally, a random subset of 500 orders was evaluated by manual review for concordance between infectious source selected and infectious source documented in the chart. Concordance was evaluated at the time of order signature. Two pharmacists performed an independent chart review, and a third pharmacist was utilized to adjudicate any discrepancies.

## Results

During the study period, 57,118 antimicrobial orders were placed. Orders with prophylaxis indication (N = 11,083) were excluded from the assessment. Infectious source was selected on 46,035 orders and included in analysis. Three percent of orders had more than one infectious source selected. Physician ordering accounted for 64% of orders, advanced practice providers accounted for 29%, and pharmacists accounted for 7%. Most orders (51%) originated from the emergency department, with 36% from regular nursing floor units, and 13% from intensive care units.

The primary outcome results of infectious source prevalence from 46,035 orders are shown in Table 1. The five most common infectious sources were respiratory (23%), urinary/renal (19%), skin/soft tissue (16%), intra-abdominal (12%), and source unknown (10%).

**Table 1.** Infectious source prevalence

	Total population (%)	Empiric indication (%)	Pathogen-directed indication (%)
Number of orders*	n = 46,035	n = 40,333	n = 5,702
*Respiratory*	10,742 (23.3%)	9,949 (24.7%)	793 (13.9%)
*Urinary/Renal*	8,535 (18.5%)	7,192 (17.8%)	1,343 (23.6%)
*Skin/Soft Tissue*	7,500 (16.3%)	6,781 (16.8%)	719 (12.6%)
*Intra-Abdominal*	5,637 (12.2%)	5,155 (12.8%)	482 (8.5%)
*Source Unknown*	4,702 (10.2%)	4,661 (11.6%)	41 (0.7%)
*Bloodstream*	2,568 (5.6%)	1,518 (3.8%)	1,050 (18.4%)
*Ears, Nose, and Throat*	1,750 (3.8%)	1,619 (4.0%)	131 (2.3%)
*Bone and Joint*	1,633 (3.5%)	1,216 (3.0%)	417 (7.3%)
*Other (Free-Text)*	1,178 (2.6%)	952 (2.4%)	226 (4.0%)
*Sexually Transmitted*	1,017 (2.2%)	972 (2.4%)	45 (0.8%)
*Obstetric/Gynecologic*	618 (1.3%)	545 (1.4%)	73 (1.3%)
*C. difficile*	526 (1.1%)	32 (0.1%)	494 (8.7%)
*Central Nervous System*	469 (1.0%)	406 (1.0%)	63 (1.1%)
*Endocarditis/Cardiac Device*	332 (0.7%)	160 (0.4%)	172 (3.0%)
*Febrile Neutropenia*	226 (0.5%)	222 (0.6%)	4 (0.1%)

* Of the 46,035 orders, there were an additional 1,398 infectious source instances due to multiple infectious sources chosen for some of those orders.

“Other” infectious source was selected in 1,178 of 46,035 (3%) orders, requiring providers to free-text an infectious source. Seventy-six percent (886/1,178) of the free-texted infectious sources could have been a prespecified infectious source. The most common free-texted infectious source was dental/oral at 277 of 1,178 (24%).

In the random sample of 500 orders from the 46,035 orders, concordance of infectious source selected to the infectious source documented in the chart was 95% (475/500). Of the 25 orders not deemed concordant, 17 were discrepant, 5 did not have evidence of an infection in the chart, and 3 did not have infectious source documented by provider in the chart.

## Discussion

This quality improvement project evaluated a mandatory infectious source selection upon ordering systemic antimicrobials to track ASP practices at a health system level. We described the prevalence of infectious sources and observed 95% concordance between documented and provider-selected sources, likely attributable to the order design requiring cascading source selection following indication choice.

Saini et al. evaluated accuracy from 14 studies regarding how well the indication chosen matched diagnosis documented in the chart.^5^ The median reported accuracy was 78% compared to our concordance rate of 95%. Within that study, the highest concordance rates were Beardsley et al.^6^ with 89.4% and Heil et al.^7^ with 90%. Our concordance rate may be higher than previously published due to the simplified terminology in the order questions and provider familiarity with order questions, which has been incorporated into our vancomycin dosing service consult since 2011.

In our analysis, most of the free-text orders could fit under a current infectious source option. Free-text sources were typically more detailed than the current infectious source options, such as free-texting chronic obstructive pulmonary disease when respiratory could have been selected. Dental/oral was identified as an infectious source to add due to its high prevalence. We did not find that the free-text option was used to bypass the hard stop.

This evaluation was conducted at a single health system, so generalizability to other settings may be limited. However, emergency departments, community hospitals, and large academic medical centers were included in the analysis. Chart review evaluated infectious source concordance and not appropriateness of the therapy, thus limiting our results to assessing the accuracy of the infectious source order question. The data were reviewed over a one-month period, which did not allow for the use of the tool over time. It would be beneficial to analyze data over the course of six months to a year to assess trends in concordance and seasonality.

The ASP Committee supported and implemented this step in antimicrobial ordering. This project shows that prescribers chose the infectious source that aligns with chart documentation, allowing the healthcare team to better evaluate accurate trends in prescribing practice. Our next step is to create a dashboard aiding our institution to evaluate prescribing patterns based on infectious sources. With a concordance rate of 95%, we are incorporating infectious source into the antimicrobial days of therapy assessments and dashboards to evaluate treatment guideline adherence and monitor trends. Prospective audit and feedback ASP alerts utilizing infectious source will be added.

## Conclusion

Implementation of a mandatory infectious source on antimicrobial orders was evaluated at a health system. The concordance rate of 95% validated that providers accurately chose the same infectious source as documented in the chart. A dashboard will be created to allow auditing in real time to help identify ASP trends and create new best practice recommendations.
